# PUF-8 Functions Redundantly with GLD-1 to Promote the Meiotic Progression of Spermatocytes in *Caenorhabditis elegans*

**DOI:** 10.1534/g3.115.019521

**Published:** 2015-06-10

**Authors:** Agarwal Priti, Kuppuswamy Subramaniam

**Affiliations:** * Department of Biological Sciences & Bioengineering, Indian Institute of Technology – Kanpur, Kanpur 208016, India; † Department of Biotechnology, Indian Institute of Technology – Madras, Chennai 600036, India

**Keywords:** GLD-1, PUF-8, meiotic differentiation, dedifferentiation, germ cell tumor

## Abstract

Successful meiotic progression of germ cells is crucial for gametogenesis. Defects in this process affect proper genetic transmission and sometimes lead to tumor formation in the germline. In *Caenorhabditis elegans*, the RNA-binding protein GLD-1 is essential for the meiotic development of oocytes. However, its role during spermatogenesis has not been understood. Here, we show that GLD-1 functions redundantly with the PUF family protein PUF-8 to ensure proper meiotic development of spermatocytes. When grown at 20°—the standard laboratory temperature for *C. elegans* growth—primary spermatocytes in both *gld-1* and *puf-8* single-mutant males and hermaphrodites complete the meiotic divisions normally. By contrast, some of the *gld-1*; *puf-8* double-mutant spermatocytes exit meiosis and form germ cell tumors in both sexes. During larval development, *gld-1*; *puf-8* double-mutant germ cells begin to express the meiotic marker HIM-3, lose P granules, and form the sperm-specific membranous organelle, which are characteristics of developing spermatocytes. However, some of these cells quickly lose HIM-3 and form germ cell tumors that lack membranous organelle but contain P granules. Mutations that block meiotic progression at late pachytene or diakinetic stage fail to arrest the tumorigenesis, suggesting that the *gld-1*; *puf-8* double-mutant spermatocytes exit meiosis prior to the completion of pachytene. Together, results presented here uncover a novel function for *gld-1* in the meiotic development of spermatocytes in both hermaphrodites and males.

Germ cells have the unique ability to switch from the mitotic to the meiotic mode of cell division. Surprisingly, in *Caenorhabditis elegans*, germ cells that have progressed significantly through the meiotic program are still capable of mitotic proliferation (Francis *et al.* 1995a; Subramaniam and Seydoux 2003). For example, *C. elegans* male germ cells missing the RNA-binding protein PUF-8 enter the meiotic program but fail to progress beyond the diakinetic stage. Instead, they dedifferentiate into germ cell tumors (Subramaniam and Seydoux 2003). Presently, it is not clear whether the dedifferentiation of developing gametes is a consequence of certain unknown defects in meiotic progression or to the result of inappropriate expression of mitosis-promoting factors. Regardless of the cause, the dedifferentiation phenotype clearly indicates that meiotic germ cells can return to the mitotic mode at least during prophase I. The significance of why these cells retain mitotic potential and the mechanism(s) that suppress this potential are largely unknown.

In *puf-8* mutant worms, spermatocytes dedifferentiate in to germ cell tumors only at elevated temperatures. When grown at 20°—the standard laboratory temperature for *C. elegans* growth—these worms do not develop germ cell tumors. In contrast, their germlines become tumorous when grown at 25°, a temperature at which wild-type germlines are still normal (Subramaniam and Seydoux 2003). Even worms homozygous for alleles that delete most of the PUF-8−coding region do not develop germ cell tumors at 20°. Thus, PUF-8 is crucial for spermatogenesis only at greater temperatures.

PUF proteins control the translation of their target mRNAs. A large number of mRNAs have been identified as potential PUF targets in yeast, worms, flies, and human (Gerber *et al.* 2004, 2006; Galgano *et al.* 2008; Morris *et al.* 2008; Kershner and Kimble 2010; Mainpal *et al.* 2011). However, PUF-mediated translational regulation in the actual biological context has not been demonstrated for many of the potential PUF targets. Results from several studies indicate that *puf-8* functions redundantly with many other genes (Bachorik and Kimble 2005; Ariz *et al.* 2009; Racher and Hansen 2012; Vaid *et al.* 2013). Thus, in addition to identifying the mRNA targets of PUF-8, identification of its redundant genetic interactors is equally important for a comprehensive understanding of how PUF-8 controls germ cell development.

Current evidences indicate that PUF-8 promotes meiotic progression only in male germ cells. Germlines of feminized hermaphrodites—these form oocytes but no sperm—missing PUF-8 are not tumorous even at 25° and form functional oocytes (Subramaniam and Seydoux 2003). Previous studies have shown that the female germ cells missing GLD-1, another translational regulator, exit meiosis prematurely and form germ cell tumors (Francis *et al.* 1995a,b). Because genetically masculinized hermaphrodites and males grown at 20° do not form germ cell tumors, the role of GLD-1 during the meiotic progression of male germ cells has not been recognized previously. We examined the *gld-1* mutant males grown at 25° and investigated the genetic interaction between *gld-1*and *puf-8*. We found that some of the *gld-1(-)* males do develop germ cell tumors when raised at 25°, which is strikingly similar to the *puf-8* mutant. In addition, in the absence of both GLD-1 and PUF-8, germ cells exit meiosis and form tumors in all males and masculinized hermaphrodites even when they are grown at 20°. These results clearly show that GLD-1 and PUF-8 function redundantly to promote the meiotic progression of male germ cells in both males as well as hermaphrodites.

## Materials and Methods

### *C. elegans* strains

All strains used in this study were maintained as described (Brenner 1974). However, transgenic lines were maintained at 25° to prevent silencing of the germline expression (Strome *et al.* 2001). Transgenes were introduced into the *puf-8(-)* background with the use of standard genetic techniques. To avoid the potential influence of the marker alleles on the phenotype, the marker genetic backgrounds were kept identical among the different strains. For example, to compare the phenotypes of *puf-8(-)* and *gld-1(-)*; *puf-8(-)*, we examined the germlines of *puf-8(zh17) unc-4(e120)*; *dpy-5(e61)* and *puf-8(zh17) unc-4(e120)*; *gld-1(q485) dpy-5(e61)*. To rule out allele-dependency of phenotypes, we repeated all experiments using two additional alleles of *puf-8*, namely *ok302* and *q725*, and did not see any significant variations among the three alleles (Table 1 and data not shown). To rule out marker-effects [*dpy-5(e61*)] on the *gld-1* phenotype, we repeated the experiments using RNA interference (RNAi)-based depletion of GLD-1 (Table 1 and Table 2, and data not shown). Strains used in this study are listed in Supporting Information, Table S1. Details on the generation of males of various genotypes are given in File S1.

**Table 1:** *gld-1* and *puf-8* function redundantly to suppress tumor formation in the male germline

Genotype	Percent of Males With Tumorous Germline	Total Number of Animals
Males grown at 20°		
* gld-1(q485)*	0	116
* puf-8(zh17)*	0	62
* gld-1(q485)*; *puf-8(zh17/+)*	25	40
* gld-1(q485)*; *puf-8(zh17)*	100	87
* gld-1(q485)*; *puf-8(zh17)*; *spe-6(hc49)*	100	21
* gld-1 (RNAi)*; *puf-8(zh17)*	100	110
* gld-1 (RNAi)*; *puf-8(q725)*	81	104
* gld-1 (RNAi)*; *puf-8(ok302)*	100	40
* rrf-1(ok589) gld-1 (RNAi)*; *puf-8(zh17)*	94	113
* rrf-1(ok589) gld-1 (RNAi)*; *puf-8(ok302)*	99	93
* spe-6(hc49)*	0	46
Males grown at 25°		
* puf-8(zh17)*	47	203
* gld-1(q485)*	51	154
* gld-1(RNAi)*	48	150
* gld-1(q485)*; *spe-6(hc49)*	10	134
* spe-6(hc49)*	0	36

See Table S1 for the complete description of the genotypes, which includes the marker mutations. For example, the complete genotype for *gld-1(q485)* listed above is *dpy-5(e61) gld-1(q485)* I; *unc-4(e120)* II; and for *puf-8(zh17)* listed above, the complete genotype is *dpy-5(e61)* I; *puf-8(zh17) unc-4(e120)* II.

**Table 2:** *gld-1* and *puf-8* function redundantly to suppress the development of spermatogenesis-dependent tumor in hermaphrodites

Genotype	Percent of Worms With Tumorous Germline	Total Number of Worms
Hermaphrodites grown at 20°		
*gld-1(q485)^a^*	100*^a^*	194
* puf-8(zh17)*	0	118
* gld-1(q485)*; *puf-8(zh17)*	100	134
* gld-1 (RNAi)*; *puf-8(q725)*	100	48
* gld-1 (RNAi)*; *puf-8(ok302)*	100	72
* gld-1(RNAi)*; *puf-8(RNAi)*	100	288
* rrf-1(ok589) gld-1 (RNAi)*; *puf-8(RNAi)*	100	122
* gld-1(q485)*; *puf-8(zh17)*; *spe-6(hc49)*	100	208
* spe-6(hc49)*	0	92
* gld-1(RNAi) mek-2(q425)*; *puf-8(RNAi)*	100	174
* gld-1(q485) mek-2(RNAi)*; *puf-8(zh17)*	100	44
* gld-1(RNAi)*; *puf-8(RNAi)*; *spe-6(hc49)*	100	174
Hermaphrodites grown at 25°		
* puf-8(zh17)*	43	181
* puf-8(RNAi)*	42	84
* rrf-1(ok589)*; *puf-8(RNAi)*	45	102
* gld-1(q485/+)*; *puf-8(zh17)*	94	147
Effect of masculinization		
* puf-8(zh17)*; *fem-3(q20)*, grown at 20°	0	324
* gld-1(RNAi)*; *puf-8(zh17)*; *fem-3(q20)*, grown at 20°	100	204
* gld-1(q485)*; *fem-3(q20)*, grown at 20°	1	260
*gld-1(q485)*; *fem-3(q20)*, grown at 15°*^a^*	100*^a^*	72

* Germline tumor in these worms was the gld-1-type. See Results and Figure 3 for more details. See Table S1 for the complete description of the genotypes, which includes the marker mutations.

### Immunostaining and fluorescence microscopy

Gonads were extruded and stained with antibodies against HIM-3, membranous organelle (MO), PH3, and P granules and the DNA-binding dye DAPI as described previously (Ariz *et al.* 2009). For the Hoechst staining reported in Figure S2, dissected gonads were incubated in 5 µM Hoechst dye (Hoechst 33343; Invitrogen) in dark for 2 hr, followed by washing with M9 buffer. Gonads were mounted on agar pad for imaging. Immunostaining using anti-RME-2 antibodies was performed as described previously (Grant and Hirsh 1999; Hansen *et al.* 2004b). Fluorescence images were acquired using a Carl Zeiss M2 microscope and the Axiovision software package following manufacturer’s protocol (Carl Zeiss). The optimum exposure conditions were determined using the wild-type genotype and the same conditions were used to image the mutants. All images presented are representative of at least 30 gonads per experiment, and each experiment was repeated at least four times.

### RNAi

Target sequences were amplified by polymerase chain reaction from cDNA from total RNA and ligated to the pSV2 RNAi feeding vector using the TA cloning method (Mainpal *et al.* 2011). The resulting RNAi constructs were introduced into the *Escherichia coli* strain HT115(DE3) and lawns prepared using the transformed bacteria were fed to worms for inducing RNAi response (Timmons *et al.* 2001).

## Results

### *gld-1* and *puf-8* function redundantly to prevent tumorigenesis in the male germline

To test whether *gld-1* and *puf-8* interact genetically, we generated a strain carrying *gld-1(q485)* and *puf-8(zh17)* alleles and examined the meiotic progression in animals homozygous for both these alleles (see the section *Materials and Methods* and Table S1). For convenience, we refer to this genotype as *gld-1(-)*; *puf-8(-)* or *gld-1*; *puf-8* in this paper. Both these alleles have been previously shown to be null alleles (Francis *et al.* 1995a; Ariz *et al.* 2009). To rule out allele-specific effects, we repeated the experiments by RNA-mediated depletion of GLD-1 in worms homozygous for the *puf-8(q725)* or *puf-8(ok302)* allele. These two also have been shown to be null alleles of *puf-8* (Subramaniam and Seydoux 2003; Bachorik and Kimble 2005).

**Figure 1:**
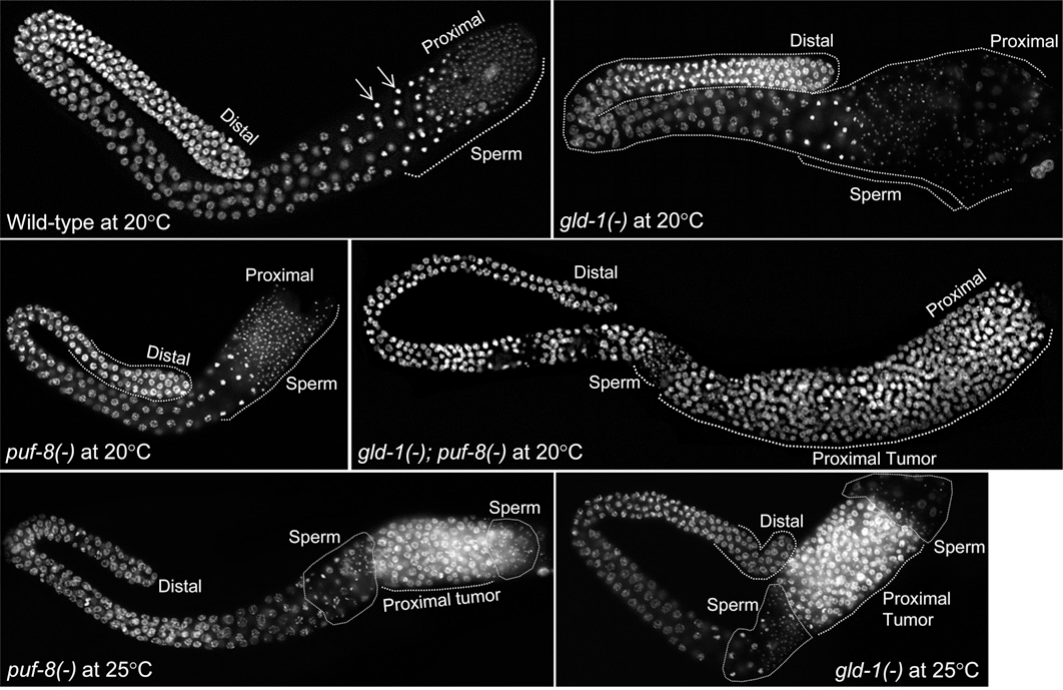
*gld-1* and *puf-8* function redundantly to suppress tumorigenesis in the male germline. Dissected male gonads of the indicated genotypes stained with the DNA-binding dye DAPI. Only the germline nuclei are visible. These males have been raised at the indicated temperatures and were 1-d-old adults at the time of dissection. Primary spermatocytes and spermatids are seen at the proximal part in the wild-type, *puf-8(-)*, and *gld-1(-)* males grown at 20°. However, all the *gld-1*; *puf-8* double-mutant germlines contain a large population of proliferating germ cells proximal to the region containing a few sperm. Similar tumors are present in approximately 51% of the *gld-1(-)* and 47% of the *puf-8(-)* males grown at 25° (Table 1). Arrows point to representative primary spermatocytes. Full description of the genotypes: *gld-1(-)* = *dpy-5(e61) gld-1(q485)* I; *unc-4(e120)* II; *puf-8(-)* = *dpy-5(e61)* I; *puf-8(zh17) unc-4(e120)* II; and *gld-1(-)*; *puf-8(-)* = *dpy-5(e61) gld-1(q485)* I; *puf-8(zh17) unc-4(e120)* II. Unless otherwise mentioned, these genotypic descriptions apply to the images shown in the subsequent figures as well.

Both *C. elegans* male and hermaphrodite germlines display distal-proximal polarity with respect to germ cell development. Undifferentiated proliferating germ cells are found at the distalmost part. As cells migrate proximally, they enter meiosis and progress through the different stages of meiosis, and gametogenesis, and form mature gametes at the proximal end of the gonad. In *puf-8(-)* males grown at 25°, germ cells enter meiosis at the normal time and space; however, some meiotic cells return to the proliferative mode without completing the meiotic divisions and form a population of proliferating cells in the proximal gonad. The other meiotic cells complete the meiotic program and form mature sperm (Subramaniam and Seydoux 2003). These mature sperm accumulate on the distal side of the proliferating-population, which expands proximally. A few mature sperm are often interspersed among the proliferating cells, and found on the proximal side of the tumor as well (Figure 1).

Consistent with the earlier observations, neither *puf-8* nor *gld-1* single-mutant males developed germ cell tumors when grown at 20°. By contrast, all *gld-1(-)*; *puf-8(-)* males developed tumors in the proximal germline (Figure 1 and Table 1). As seen in *puf-8(-)* males raised at 25°, a few mature sperm did accumulate on the distal side of the tumor in *gld-1(-)*; *puf-8(-)* male germlines, indicating that some meiotic cells complete spermatogenesis in these germlines as well. However, perhaps because fewer cells successfully complete meiosis when both GLD-1 and PUF-8 are absent, we could not readily observe mature sperm interspersed among the tumor cells, or on the proximal side of the tumor in these germlines (Figure 1). Immunostaining for the germ cell-specific P granules confirmed that the proliferating cells in the proximal germline were indeed germ cells (Figure S1).

Because some of the *puf-8(-)* males form germ cell tumors when grown at 25° (Subramaniam and Seydoux 2003), we grew the *gld-1(-)* males at 25° to check whether they also formed similar tumors at this temperature. Approximately 51% of *gld-1(-)* males grown at 25° developed germ cell tumors, which was strikingly similar to that observed in *puf-8(-)* males (Figure 1 and Table 1). Although no specific data have been published, tumor formation in *gld-1* mutant males at 25° has been observed by others as well (see the legend to Figure 3 in Ciosk *et al.* 2004).

**Figure 2:**
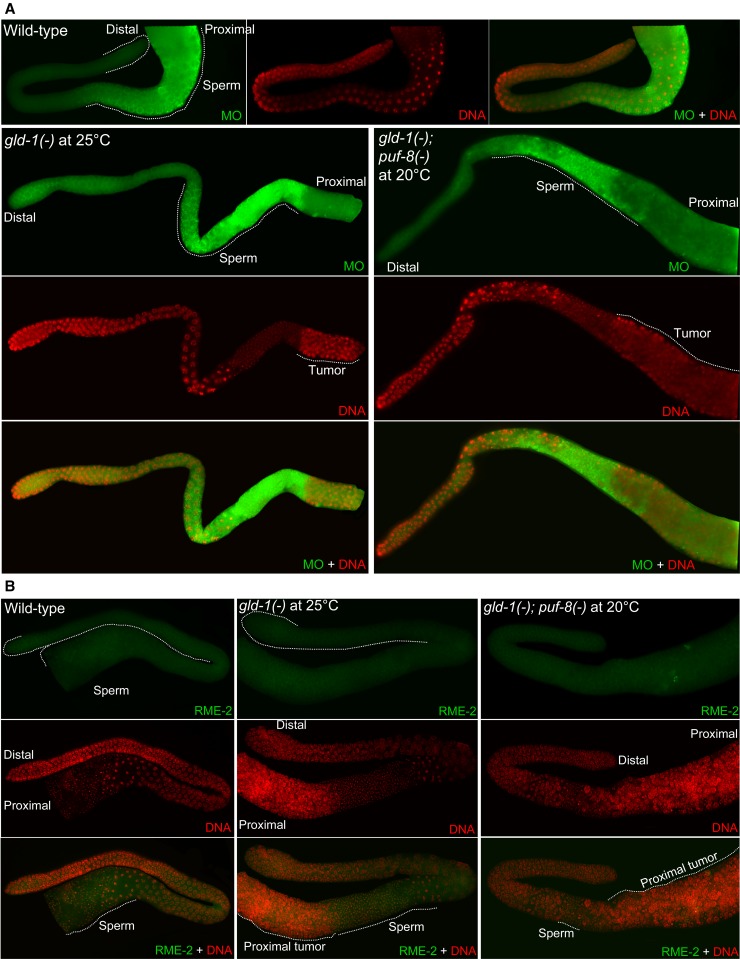
Specification of the male sexual fate of germ cells does not depend on *gld-1* or *puf-8*. (A) Germlines of males, of the indicated genotypes and growth temperatures, stained with anti-membranous organelle (MO) antibodies (green) and DAPI (red). Cells positive for the sperm marker MO—outlined and marked sperm—are present on the distal side of the tumor in both *gld-1(-)* and *gld-1(-)*; *puf-8(-)* germlines. (B) Male germlines stained for the oocyte marker RME-2 (green) and DAPI (red). No RME-2−positive cells are seen in any of the three genotypes shown. For a positive control of RME-2 immunostaining, see Figure 4B.

One possibility is that the germ cells of *gld-1(-)* males grown at 25° and *gld-1(-)*; *puf-8(-)* males grown at 20° fail to enter spermatogenesis and instead switch to the oogenic mode. Female germ cells thus formed in the male germline probably exit meiosis and form tumors, like their counterparts in *gld-1(-)* hermaphrodites (Francis *et al.* 1995b). To test this possibility, we examined the germlines of these males for the presence of RME-2, an oocyte-specific yolk receptor, and the sperm-specific MO by immunostaining with specific antibodies (Ward *et al.* 1986; Grant and Hirsh 1999). Both *gld-1(-)* and *gld-1(-)*; *puf-8(-)* male germlines did not express RME-2, but stained positively for MO (Figure 2), which shows that the germ cells did not switch to oogenic mode in these germlines. *rme-2* mRNA is a well-known target of GLD-1, and RME-2 has been shown to be misexpressed in the pachytene region of *gld-1(-)* hermaphrodite germlines (Lee and Schedl 2001). However, the absence of RME-2 in male germlines missing GLD-1 is not surprising because the suppression of RME-2 expression in male germlines has been shown to be independent of GLD-1 (Ciosk *et al.* 2004). Together, the aforementioned data show that *puf-8* and *gld-1* function in a redundant fashion to suppress tumorigenesis in the male germline.

On the basis of the expression pattern of a PUF-8::GFP fusion, which rescues the germline defects of *puf-8* mutations, PUF-8 is thought to be expressed predominantly in the distal germline of hermaphrodites (Ariz *et al.* 2009; Racher and Hansen 2012). We observed a similar expression pattern in males as well with the *puf-8*::*gfp* transgene described by Ariz *et al.* (Ariz *et al.* 2009) (Figure S2). To determine whether the expression of PUF-8 in germ cells is essential for normal spermatogenesis, we depleted PUF-8 by RNAi in the *rrf-1* mutant [*rrf-1(ok589)*], which is known to restrict the RNAi effect to germ cells (Smardon *et al.* 2000; Sijen *et al.* 2001). Similar to *puf-8(zh17)* males, *rrf-1(ok589)*; *puf-8(RNAi)* males grown at 25° also developed tumors in the germline (Table 1), indicating that the activity of PUF-8 is indeed required in the germ cells to prevent tumorigenesis.

### *gld-1* and *puf-8* function redundantly to suppress the spermatogenesis-dependent tumor formation in hermaphrodites

**Figure 3:**
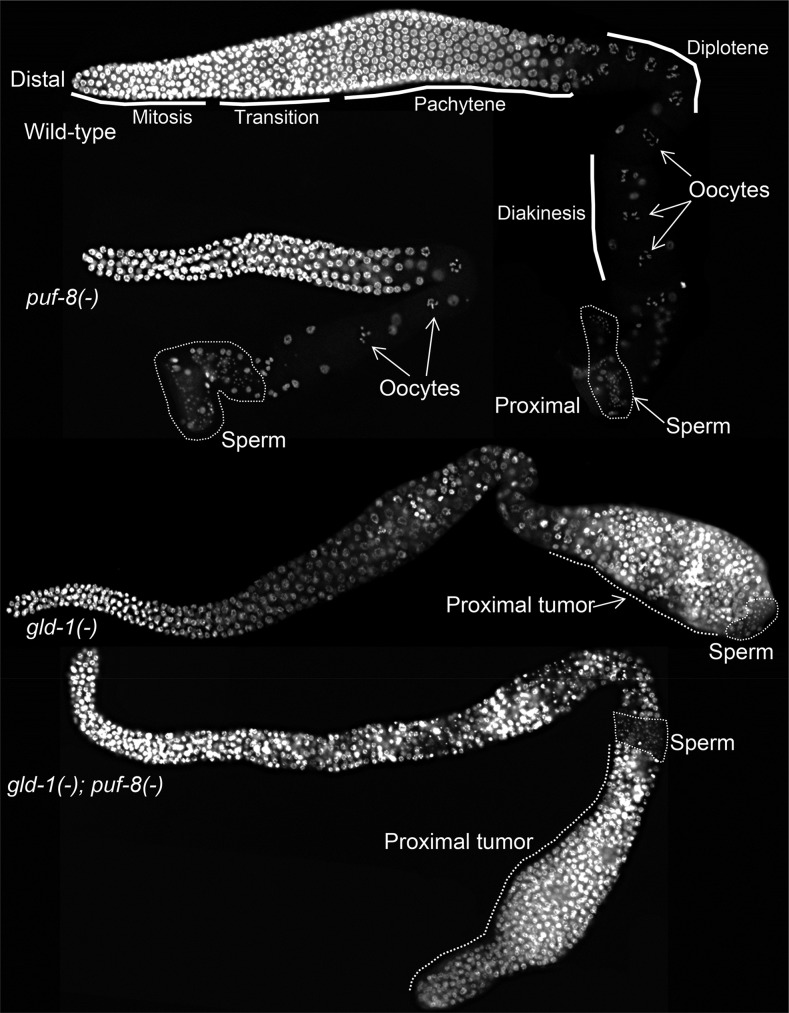
Comparison of germ cell tumors between *gld-1(-)* and *gld-1(-);puf-8(-)* hermaphrodite germlines. Hermaphrodite gonads of the indicated genotypes stained with DAPI. Mitotically cycling population of germline stem cells is present at the distal end of the gonad. Successive temporal stages of differentiating germ cells are present consecutively in the distal-to-proximal axis with the fully-developed gametes at the proximal end. Sperm nuclei appear as tiny dots within the marked area. Whereas the tumor is on the distal side of sperm in the *gld-1* single mutant, it is on the proximal side of sperm in the *gld-1*; *puf-8* double mutant.

During larval development, the first few germ cells that enter meiosis in hermaphrodites differentiate as sperm. In approximately 50% of *puf-8(-)* hermaphrodites raised at 25°, spermatocytes dedifferentiate into germ cell tumors (Subramaniam and Seydoux 2003). The *gld-1* and *puf-8* functional redundancy that we saw in males prompted us to ask whether *gld-1* functioned redundantly with *puf-8* in hermaphrodites as well during spermatogenesis. As mentioned previously, in *gld-1(-)* hermaphrodites, tumors originate from female germ cells that fail to progress through meiosis (Francis *et al.* 1995a,b). Therefore, to address the above question, it is necessary to distinguish between tumors arising from male (puf-8-type) and female (gld-1-type) germ cells. Similar to *puf-8(-)* males, a cluster of sperm is present on the distal side of the tumor in *puf-8(-)* hermaphrodites. In contrast, because spermatogenesis is completed in *gld-1* mutant hermaphrodites before the onset of tumorigenesis, sperm are present only on the proximal side of the tumor (Figure 3). Staining with DAPI revealed that all *gld-1*; *puf-8* double-mutant hermaphrodites developed the puf-8-type tumor even when grown at 20° (Figure 3 and Table 2). None of these worms had sperm on the proximal side of the tumor. At this temperature, consistent with the earlier observations, none of the *puf-8(-)* hermaphrodite germlines became tumorous, and all *gld-1(-)* hermaphrodites formed only the gld-1-type tumor (Figure 3 and Table 2). These results suggest that the removal of *gld-1* activity enhances the *puf-8* mutant phenotype in hermaphrodites.

**Figure 4:**
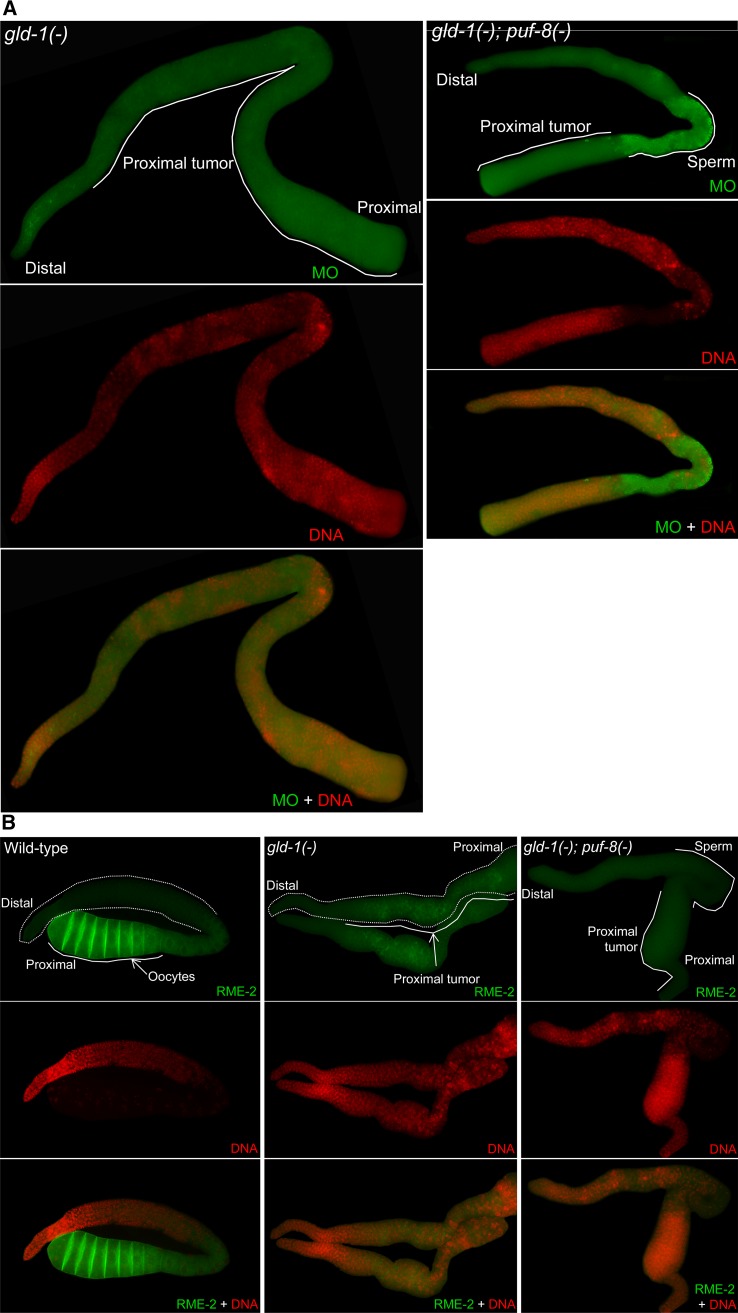
Expression patterns of sperm- and oocyte-specific markers in the *gld-1(-)* and *gld-1(-)*; *puf-8(-)* hermaphrodite germlines. The gonads of hermaphrodites of the indicated genotypes were dissected out and stained with (A) anti-membranous organelle (MO) antibodies (green) and DAPI (red), or (B) anti-RME-2 (green) and DAPI (red). Whereas the *gld-1(-)* germlines have stained positively for RME-2, MO-specific staining is undetectable in them. In contrast, the *gld-1(-)*; *puf-8(-)* germlines show an opposite expression pattern.

To further confirm the aforementioned observations, we compared *gld-1(-)* and *gld-1(-)*; *puf-8(-)* hermaphrodite germlines after immunostaining with anti-MO and anti-RME-2 antibodies. As shown in Figure 4, *gld-1* single mutant germlines stained positively for RME-2, but not for MO. By contrast, in *gld-1(-)*; *puf-8(-)* germlines, the sperm-like nuclei revealed by DAPI-staining were all MO-positive, and no RME-2-positive cells were observed. Additionally, P granules were present continuously throughout the *gld-1(-)* germline, whereas they were not detected in the sperm-rich region of the *gld-1(-)*; *puf-8(-)* germline (Figure S3). Nevertheless, these results do not establish that the tumor in the double-mutant hermaphrodite germline was actually dependent on spermatogenesis. Loss of *puf-8* function has been shown to masculinize the hermaphrodite germline (Bachorik and Kimble 2005; Ariz *et al.* 2009). Therefore, absence of PUF-8 could have led to the accumulation of a few sperm, and the tumor actually originated from female germ cells that did not express RME-2 for unknown reasons.

If tumors in *gld-1(-)*; *puf-8(-)* hermaphrodite germlines originate only from female germ cells, then masculinization should prevent them from forming. So, we masculinized the hermaphrodite germlines using a temperature-sensitive, gain-of-function mutation in *fem-3* [*fem-3(q20)*], a sex-determination pathway gene that promotes spermatogenesis (Barton *et al.* 1987). *gld-1(-)*; *fem-3(gf)* hermaphrodites developed germ cell tumors at the permissible temperature of 15°, at which *fem-3(q20)* mutation does not affect oogenesis. However, *gld-1(-)*; *fem-3(gf)* hermaphrodites did not develop tumors at 20°, at which *fem-3(q20)* prevents the switch from spermatogenesis to oogenesis (Table 2). This finding is consistent with the earlier conclusion that tumors in *gld-1(-)* germlines arise only from female germ cells (Francis *et al.* 1995b). *puf-8(-)*; *fem-3(gf)* hermaphrodites did not develop tumors at either temperature, which is again consistent with the earlier observation that the *puf-8(-)* worms develop germ cell tumors only when grown at 25° (Subramaniam and Seydoux 2003). By contrast, all *gld-1(-)*; *puf-8(-)*; *fem-3(gf)* hermaphrodites developed germ cell tumors at 20° (Table 2), indicating that the tumorigenesis in germlines missing both GLD-1 and PUF-8 is not dependent on oogenesis.

On the basis of the results presented in the above two sections, we conclude GLD-1 functions redundantly with PUF-8 to suppress the formation of tumors arising from male germ cells in both males as well as hermaphrodites.

### *gld-1*; *puf-8* double-mutant germ cells exit meiosis and form germ cell tumors

In *C. elegans*, germ cell tumors arise from defects at two distinct stages of development – one, mitosis-to-meiosis transition, and two, meiotic progression (Francis *et al.* 1995a,b; Berry *et al.* 1997; Kadyk and Kimble 1998; Pepper *et al.* 2003; Subramaniam and Seydoux 2003; Hansen *et al.* 2004a). Tumors arising from *puf-8(-)* male germ cells and *gld-1(-)* female germ cells, as mentioned in the Introduction, are of the latter type (Francis *et al.* 1995a; Subramaniam and Seydoux 2003). In *gld-1(-)*; *puf-8(-)* adult germlines, chromatin condensation characteristic of pachytene-stage spermatocytes began at the normal location. In addition, the sperm-specific structure MO started to form and the germ cell-specific P granules, which are normally absent in sperm, began to disappear at the same location. We saw similar expression patterns in the germlines of *gld-1(-)* males grown at 25° (Figure 2A, Figure 4A, Figure S1, and Figure S3). These observations clearly indicate that the double-mutant germ cells enter meiosis and spermatogenesis normally in adults. Nonetheless, it is possible that a few germ cells never enter meiosis, and remain quiescently in the proximal germline, and resume proliferation later.

To test the aforementioned possibility, we performed two sets of experiments. First, we blocked the meiotic progression using mutations such as *mek-2(q425*) and *spe-6(hc49)*, which prevent tumor formation from cells that prematurely exit meiosis (Subramaniam and Seydoux 2003; Vaid *et al.* 2013). Whereas 51% of *gld-1(-)* males grown at 25° developed germ cell tumors, only 10% of *gld-1(-)*; *spe-6(-)* males developed similar tumors (Table 1). Thus, germ cell tumors in *gld-1(-)* males arise largely as the result of premature meiotic exit, which is similar to what has been reported in *puf-8(-)* males (Subramaniam and Seydoux 2003). Surprisingly, all *gld-1(-)*; *puf-8(-)*; *spe-6(-)* males developed germ cell tumors even when grown at 20° (Table 1). Similarly, the removal of MEK-2 also did not stop tumor development in germlines missing both GLD-1 and PUF-8 (Table 2).

**Figure 5:**
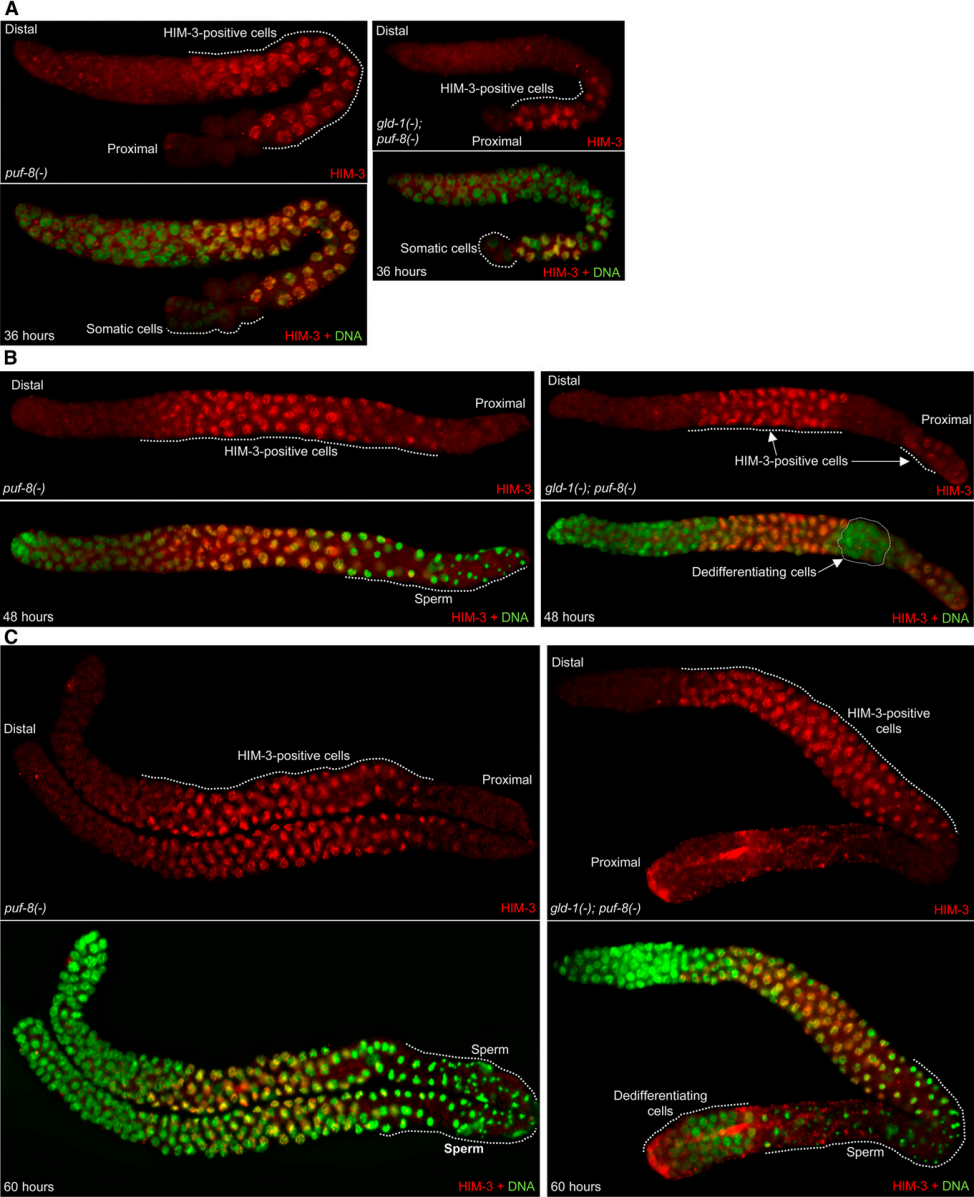
Primary spermatocytes lacking GLD-1 and PUF-8 prematurely exit meiosis. Hermaphrodites of the indicated genotypes grown at 20° and were dissected at 36 (A), 48 (B) and 60 (C) hr after hatching. The extruded germlines were stained with anti-HIM-3 antibodies (red) and DAPI (green). Germ cells at the proximal end of the germline are positive for HIM-3 in both genotypes at 36 hr. However, at 48 hr, a population of HIM-3-negative cells is readily observable amid HIM-3-positive population in all the *gld-1(-)*; *puf-8(-)* germlines (n = 50). At 60 hr, in the *gld-1(-)*; *puf-8(-)* germlines, some of the meiotic cells complete meiosis and form a few sperm, whereas the HIM-3-negative population expands at the proximal end (C, right panel). Two gonads, lying side-by-side are shown for the *puf-8(-)* single mutant. See Figure S4 as well.

Second, we dissected gonads at different stages of larval development and immunostained them for the lateral-element component HIM-3, which is commonly used as a marker for meiotic cells (Zetka *et al.* 1999). In *gld-1(-)*; *puf-8(-)* hermaphrodite germlines, all proximal-most germ cells became positive for HIM-3 at approximately 36 hr after hatching (mid L3 larva) (Figure 5A). No HIM-3-negative germ cells were found in the proximal region at this time point. Interestingly, a group of HIM-3-negative cells arose among the HIM-3-positive cells at approximately 48 hr after hatching (Figure 5B). No such HIM-3-negative cells were observed in the control [*puf-8(-)*] germlines; instead, at this time point, primary spermatocytes and spermatids were present in the proximal part of these germlines. At approximately 60 hr after hatching, the population of HIM-3-negative cells expanded at the proximal end of *gld-1*; *puf-8* double-mutant germlines. However, several sperm were present in the region between the HIM-3-positive cells and the proliferating cells at the proximal end, indicating that some germ cells successfully complete meiosis and form sperm (Figure 5C). Thus, in *gld-1(-)*; *puf-8(-)* germlines, the onset of meiosis during larval development is unaffected and no mitotic germ cell remains quiescently in the proximal region. Consistently, proliferating cells in the proximal germline did not appear before the start of HIM-3 expression. On the basis of the results of the aforementioned two sets of experiments, we conclude germ cell tumors in the *gld-1*; *puf-8* double-mutant germline originate from cells that exit meiotic development prior to the stage requiring MEK-2 or SPE-6 activity.

Cells positive for HIM-3 expression were present in *gld-1(-)* males grown at 25° and *gld-1(-)*; *puf-8(-)* males grown at 20° (Figure S4), indicating that the meiotic entry is unaffected in these worms as well. In *gld-1(-)* males, HIM-3 expression could be detected in a few cells in the proximally proliferating population, perhaps due to meiotic re-entry. Presence of HIM-3-positive cells has been previously observed in *puf-8(-)* males as well (Subramaniam and Seydoux 2003).

Germ cells in which the onset of meiosis is delayed have been shown to come under the influence of proliferation-promoting signals from somatic gonadal cells called the sheath cells, which act as a “latent niche” (McGovern *et al.* 2009). Similar to the earlier observations in *gld-1(-)* hermaphrodites (McGovern *et al.* 2009), depletion of APX-1, the primary latent niche signal, did not affect tumor formation in *gld-1(-)*; *puf-8(-)* hermaphrodites, suggesting that the tumor development in these worms is not dependent on the latent signal (Figure S5).

## Discussion

The work presented here uncovers a novel function for *gld-1* and a functional relationship between *gld-1* and *puf-8*. At the standard *C. elegans* growth temperature of 20°, formation of germ cell tumor in worms homozygous for *gld-1* null alleles is dependent on the female sex of germ cells. Based on this, previous studies had concluded that *gld-1* is essential only for the meiotic development of oocytes (Francis *et al.* 1995b). The discovery of *gld-1*’s role in the meiotic development of spermatocytes has been hampered by the strong functional redundancy between *gld-1* and *puf-8*, which the current study has uncovered. Results presented here provide unequivocal evidence for the involvement of GLD-1 in the meiotic development of spermatocytes in both hermaphrodites and males.

### Functional redundancy between *gld-1* and *puf-8*

*gld-1* has two distinct functions during gametogenesis in *C. elegans*. One, it promotes meiotic entry in both hermaphrodites and males by functioning redundantly with *gld-2* (Kadyk and Kimble 1998). Two, during meiotic progression, *gld-1* has an essential role in female germ cells (Francis *et al.* 1995a) and, as shown in the current study, a redundant function with *puf-8* in male germ cells. Previous epistasis analyses revealed that the redundant *gld-1* and *gld-2* pathways that promote meiotic entry are not dependent on *puf-8* (Racher and Hansen 2012). Thus, the functional redundancy between *gld-1* and *puf-8* is most likely limited to the promotion of spermatocyte meiotic progression.

Currently we are not sure about how *gld-1* and *puf-8* function redundantly. GLD-1 has been shown to interact with the *puf-8* 3′UTR (Beadell *et al.* 2011; Wright *et al.* 2011). Therefore, one possibility is that GLD-1 suppresses *puf-8* mRNA translation, and the overexpression of PUF-8 contributes to the premature meiotic exit in *gld-1(-)* males grown at 25°. However, this is inconsistent with the observation that the loss of PUF-8 leads to tumor formation at 25° (Subramaniam and Seydoux 2003). On the other hand, the observed expression patterns of PUF-8 and GLD-1do not support the possibility that *gld-1* promotes *puf-8* expression: PUF-8 is most abundant in the distal germline, where GLD-1 expression is low, and less abundant in the early pachytene region, where GLD-1 is most abundant (Jones *et al.* 1996; Ariz *et al.* 2009; Racher and Hansen 2012).

Because both GLD-1 and PUF-8 are known translational regulators, an alternative possibility is that their independent downstream targets function redundantly to promote spermatogenesis. As per this model, expression of either one of a GLD-1 or a PUF-8 target is sufficient to maintain the meiotic commitment in male germ cells. However, GLD-1 has not yet been shown to promote the expression of any of its targets; even a large-scale analysis did not find any mRNAs positively regulated by GLD-1 (Wright *et al.* 2011). Nevertheless, the existence of a factor positively regulated by GLD-1 cannot be excluded; therefore we do not formally rule out this model. We propose a third model in which the misexpression of a common target(s), which is controlled redundantly by GLD-1 and PUF-8, interferes in spermatogenesis. A large number of potential targets have been identified for GLD-1 and PUF-8, and several of these potential targets are common to both proteins (Mainpal *et al.* 2011; Wright *et al.* 2011). Therefore, it is probable that some of the potential common targets are either redundantly or synergistically controlled by GLD-1 and PUF-8. The product of a synergistically suppressed mRNA is likely to accumulate faster in the *gld-1*; *puf-8* double mutant than in the *gld-1* and *puf-8* single mutants, which might explain why germ cells exit meiosis at an earlier time point in the double mutant.

### Control of meiotic progression of male *vs.* female germ cells

From the results of earlier work, control of meiotic progression appeared to depend on distinct regulators in male and female germ cells (Francis *et al.* 1995b; Subramaniam and Seydoux 2003). Our current results, together with the previous findings, however, reveal that a single regulator, namely GLD-1, is sufficient to maintain meiotic commitment in both sexes. Thus, the downstream effectors and the underlying mechanism(s) are possibly shared between the two sexes. However, the meiotic progression of female germ cells requires GLD-1, but not PUF-8, which is somewhat inconsistent with the above suggestion. Differences in the expression patterns of GLD-1 and PUF-8 may, at least partly, explain this apparent inconsistency. GLD-1 is strongly expressed in the extended pachytene region of hermaphrodites, whereas the expression of PUF-8 in this region is weak (Jones *et al.* 1996; Ariz *et al.* 2009).

## 

## Supplementary Material

Supporting Information
