# Supplementary material for: PUF-8 Functions Redundantly with GLD-1 to Promote the Meiotic Progression of Spermatocytes in Caenorhabditis elegans
Source: G3 (Bethesda). 2015 Jun 10;5(8):1675–84. doi: 10.1534/g3.115.019521 (PMC4528324; doi:10.1534/g3.115.019521)
Supplement: Supporting Information [file supp_5_8_1675__index.html]

PUF-8 Functions Redundantly with GLD-1 to Promote the Meiotic Progression of Spermatocytes in Caenorhabditis elegans — Supporting Information 

# PUF-8 Functions Redundantly with GLD-1 to Promote the Meiotic Progression of Spermatocytes in *Caenorhabditis elegans*

## Supporting Information for Priti and Subramaniam, 2015

**Files in this Data Supplement:**

- Supporting Information - File S1, Figures S1-S5, Table S1, and Supplementary References (PDF, 419 KB)
- File S1 - Supplementary methods. (PDF, 153 KB)
- Figure S1 - The germ cell-specific P granules are present in the tumor cells observed in the germlines of *gld-1(-)* and *gld-1(-); puf-8(-)* males. (PDF, 389 KB)
- Figure S2 - Expression pattern of PUF-8::GFP in male germlines. (PDF, 321 KB)
- Figure S3 - Tumor cells of *gld-1(-)* and *gld-1(-); puf-8(-)* hermaphrodites contain P granules. (PDF, 366 KB)
- Figure S4 - Meiotic entry is unaffected in *gld-1(-)* and *gld-1(-); puf-8(-)* males grown at 25°C. (PDF, 347 KB)
- Figure S5 - Proximal proliferation in the *gld-1(-); puf-8(-)* mutants is not dependent on latent niche signaling. (PDF, 297 KB)
- Table S1 - *C. elegans* strains used in this study. (PDF, 151 KB)
