# Supplementary material for: PUF-8 Functions Redundantly with GLD-1 to Promote the Meiotic Progression of Spermatocytes in Caenorhabditis elegans
Source: G3 (Bethesda). 2015 Jun 10;5(8):1675–84. doi: 10.1534/g3.115.019521 (PMC4528324; doi:10.1534/g3.115.019521)
Supplement: Supporting Information [file supp_g3.115.019521_019521SI.pdf]

G3 · Genes | Genomes | Genetics

PUF-8 functions redundantly with GLD-1 to promote the meiotic progression of spermatocytes in *Caenorhabditis elegans*

Agarwal Priti[*] and Kuppuswamy Subramaniam[*, §, 1]

[*]Department of Biological Sciences & Bioengineering, Indian Institute of Technology – Kanpur, Kanpur 208016, India, [§]Department of Biotechnology, Indian Institute of Technology – Madras, Chennai 600036, India

[1]Corresponding author: Department of Biotechnology, Indian Institute of Technology – Madras, Chennai 600036, India. Email: subbu@iitm.ac.in

DOI: 10.1534/g3.115.019521

File S1

Supplementary methods

Generation of males of different genotypes

gld-1(q485), puf-8(zh17), puf-8(ok302), puf-8(q725)* and *spe-6(hc49) males were generated by mating N2 males with hermaphrodites heterozygous for the respective mutant allele and appropriate balancer (Table S1). Wild-type (heterozygous) F1 progeny males were then back-crossed with the heterozygous hermaphrodites several times until 25% of the male population were homozygous for the desired mutant allele. These males were maintained by crossing the heterozygous mutant males with the mutant hermaphrodites.

dpy-5(e61) gld-1(q485); puf-8(zh17) unc-4(e120) males were generated by mating *puf-8(zh17) unc-4(e120) / mnC1* males with IT85 [*dpy-5(e61) gld-1(q485) / hT1 I; puf-8(zh17) unc-4(e120) / mnC1* II] hermaphrodites. Wild-type F1 progeny males were then back-crossed with the mutant hermaphrodites and this process was repeated until 25% of the male population in the plate were homozygous for the desired mutant allele. Genotypes of homozygous mutant males were confirmed based on the phenotypes of the marker mutations linked to *gld-1(q485)* and *puf-8(zh17)* alleles initially and then by the tumorous germline phenotype of *gld-1(q485); puf-8(zh17)*. These males were maintained by crossing the heterozygous males with the IT85 hermaphrodites.

rrf-1(ok589); puf-8(zh17)* and *rrf-1(ok589); puf-8(ok302) males were generated by mating *puf-8(zh17) unc-4(e120) / mnC1* males and *puf-8(ok302) unc-4(e120) / mnC1* males with IT179 [*rrf-1(ok589); puf-8(zh17)*] and IT253 [*rrf-1(ok589); puf-8(ok302)*] hermaphrodites, respectively. Wild-type F1 progeny males were then back-crossed with the respective mutant hermaphrodites and this process was repeated several times to obtain homozygous mutant males. *rrf-1(ok589)* mutation was detected by PCR and the *puf-8* allele by the phenotype of the marker mutation and also by checking for the *puf-8* phenotype at 25ºC.

gld-1(q485); spe-6(hc49) males were generated by mating *dpy-5(e61) gld-1(q485) / hT1* males with IT971 [*dpy-5(e61) gld-1(q485) / hT2* I*; spe-6(hc49) unc-25(e156) / hT2* III] hermaphrodites. Wild-type F1 progeny males were then back-crossed with the respective mutant hermaphrodites and this process was repeated several times to obtain homozygous mutant males. The genotypes of homozygous mutant males were confirmed by the phenotypes of marker mutations linked to *gld-1(q485)* and *spe-6(hc49),* and also by checking for the phenotype of *gld-1(q485); puf-8(zh17)* hermaphrodites.

gld-1(q485); puf-8(zh17); spe-6(hc49) males were generated by mating *puf-8(zh17) / mC6g* males with IT958 [*dpy-5(e61) gld-1(q485) / hT2* I*; puf-8(zh17) unc-4(e120) / mnC1* II; *spe-6(hc49) unc-25(e156) / hT2* III] hermaphrodites. GFP-positive F1 progeny males were selected using a fluorescence stereomicroscope and mated with IT958 hermaphrodites and this process was repeated several times. Non-GFP males that displayed the dumpy and uncoordinated phenotypes were selected to check for the phenotype of *gld-1(q485); puf-8(zh17); spe-6(hc49)*.

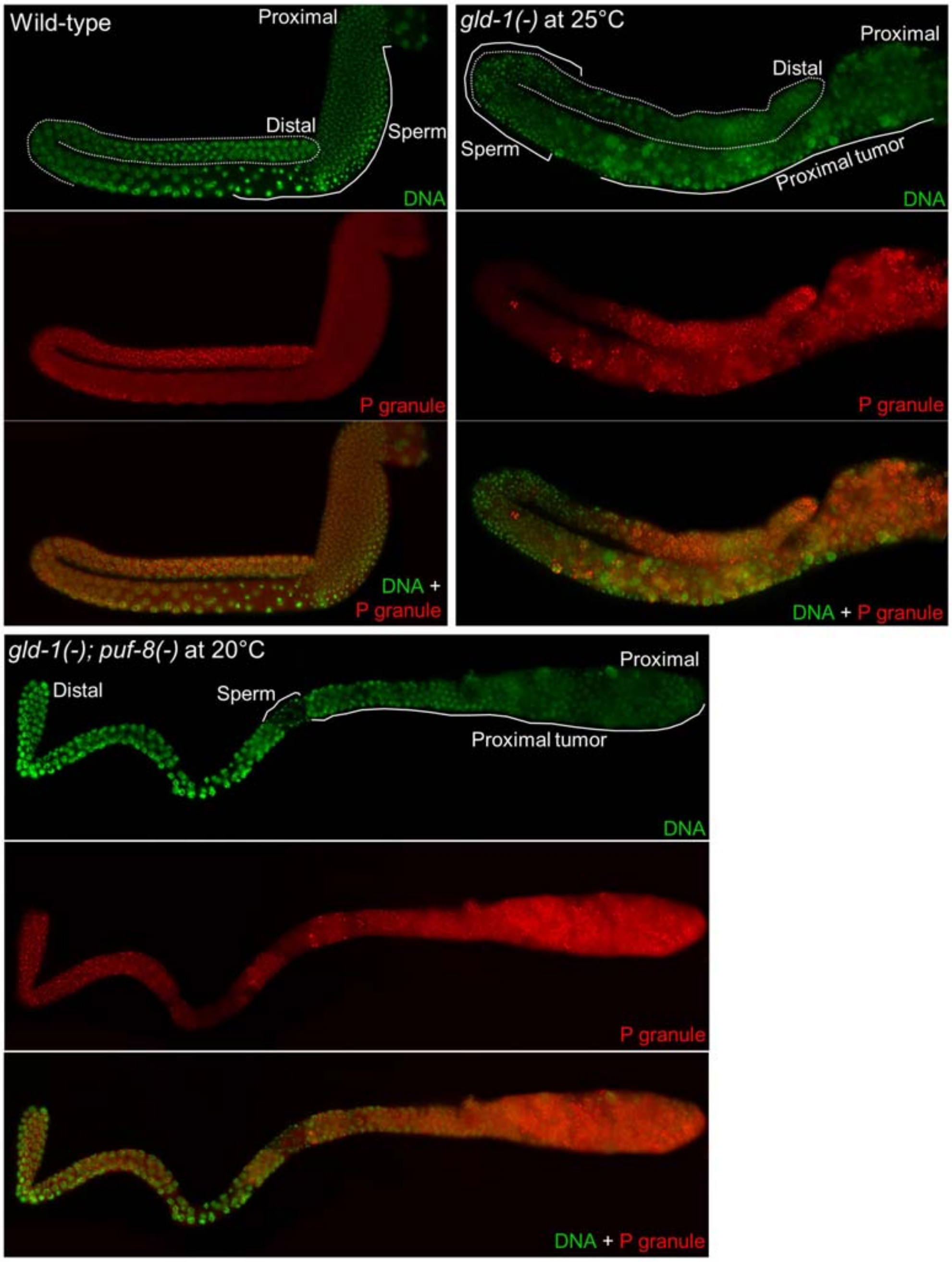

Figure S1 The germ cell-specific P granules are present in the tumor cells observed in the germlines of *gld-1(-)* and *gld-1(-); puf-8(-)* males. Dissected gonads of the indicated genotypes stained with anti-P granule antibodies and DAPI. While the P granules are not seen in the sperm present in the proximal part of wild-type gonads, they are readily visible in the proliferating cells present in the same region of the *gld-1(-)* and *gld-1(-); puf-8(-)* germlines.

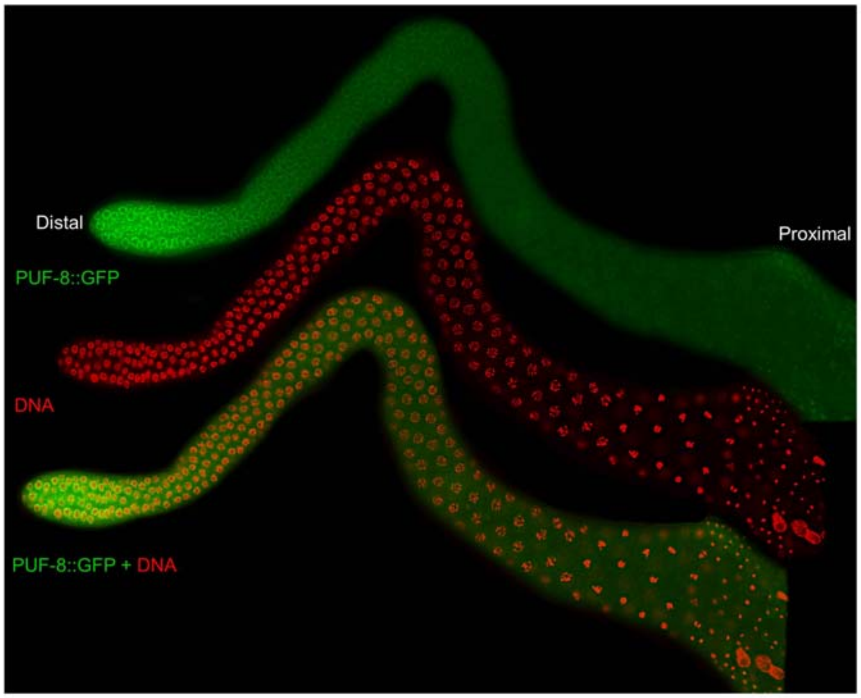

Figure S2 Expression pattern of PUF-8::GFP in male germlines. Dissected germline of a male carrying the kpIs[pMP15] transgene. This transgene expresses PUF-8::GFP fusion under the control of *puf-8* promoter and *puf-8* 3' UTR (Ariz et al. 2009). Strong expression of PUF-8::GFP (green) is seen in the distal germline, where PUF-8::GFP localization on perinuclear P granules is noticeable. DNA has been visualized by staining with Hoechst stain (red).

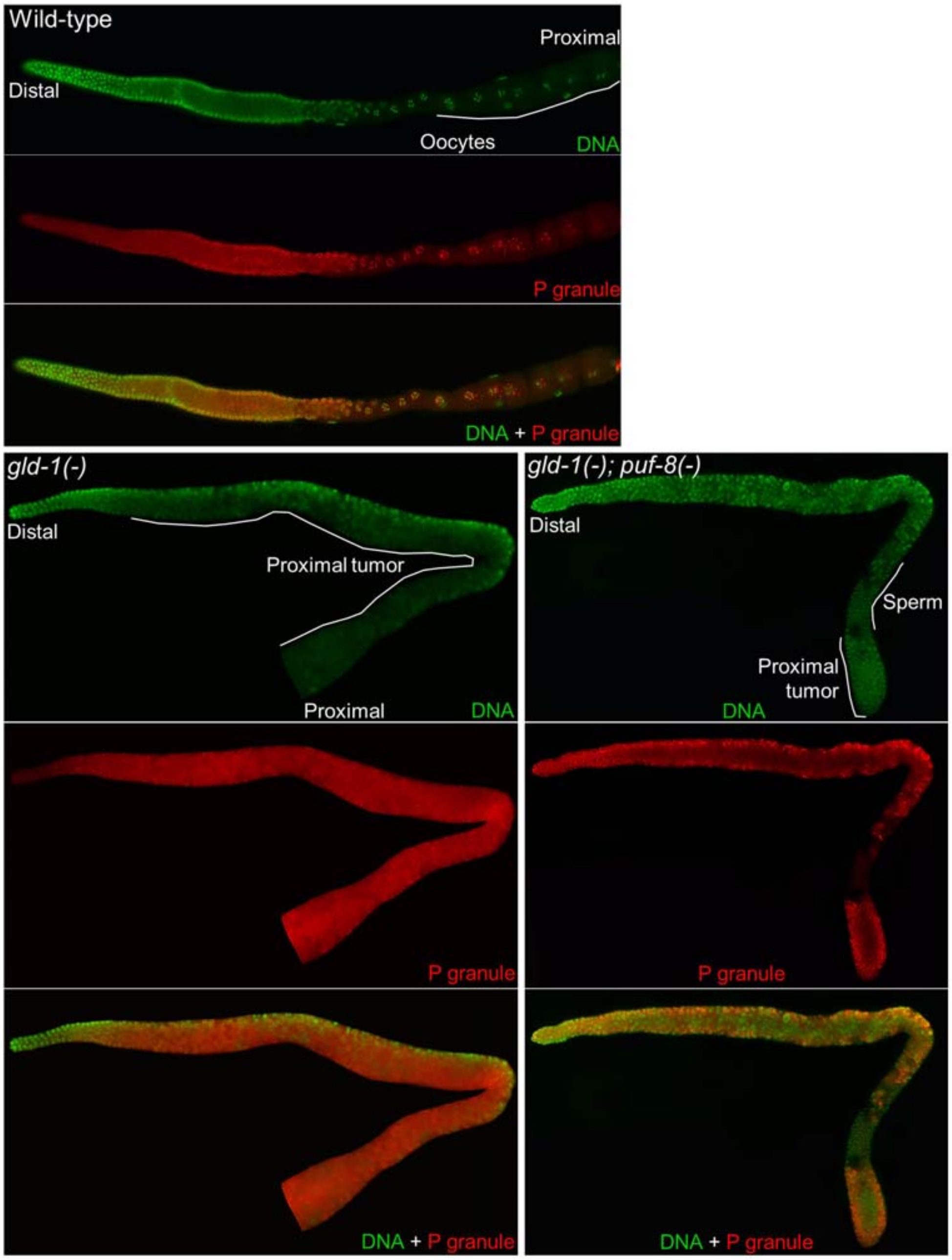

Figure S3 Tumor cells of *gld-1(-)* and *gld-1(-); puf-8(-)* hermaphrodites contain P granules. Dissected gonads of the indicated genotypes stained with anti-P granule antibodies and DAPI. In the wild-type germline, the P granules are seen in all cells including the developing oocytes. Similarly, the proximal tumor cells of *gld-1(-)* and *gld-1(-)*; *puf-8(-)* germlines as well stain positively for the germ cell-specific P granules. However, P granules are not present in the sperm seen in the *gld-1(-); puf-8(-)* germline; absence of P granules in sperm has been observed in the wild-type as well (Subramaniam and Seydoux 2003).

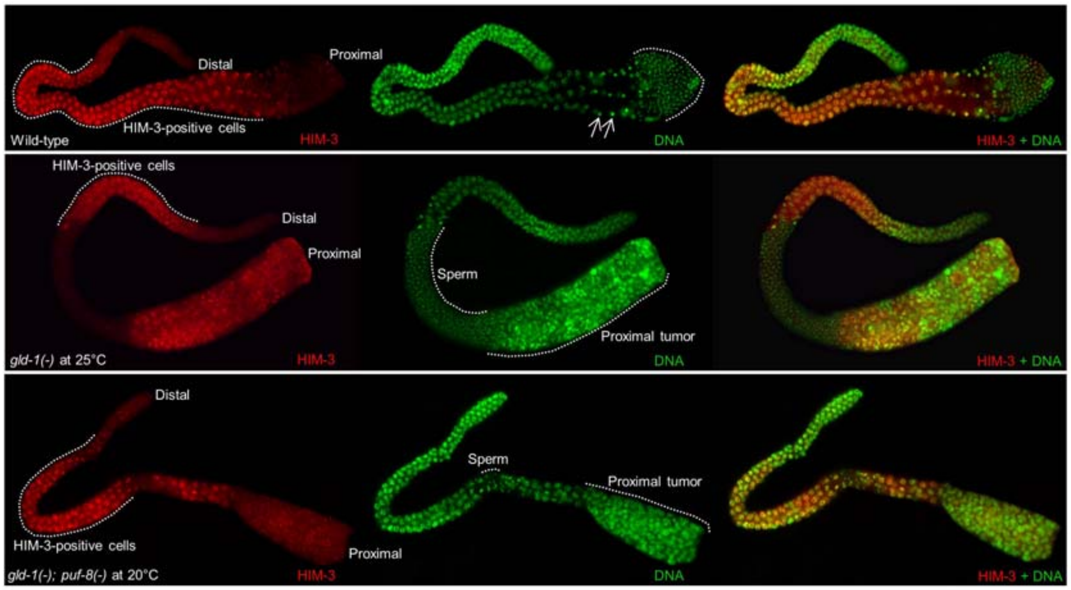

Figure S4 Meiotic entry is unaffected in *gld-1(-)* and *gld-1(-); puf-8(-)* males grown at 25°C. Gonads have been extruded out of males raised at 25°C and stained for the HIM-3 meiotic marker (red) and DAPI (green). Region of the germline containing HIM-3-positive cells have been indicated by a dashed white line in images shown on the left panel. Proximal tumors are outlined in images shown on the middle panel.

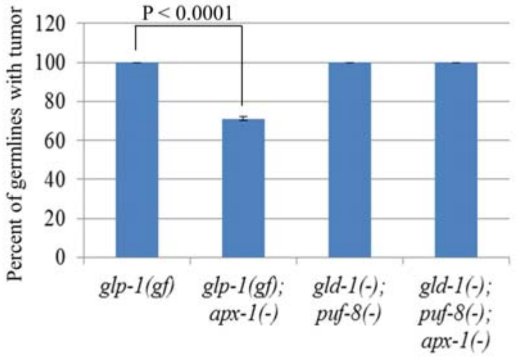

Figure S5 Proximal proliferation in the *gld-1(-); puf-8(-)* mutants is not dependent on latent niche signaling. Bar graph showing the effect of APX-1 depletion on tumor development. Worms homozygous for the *ar202* allele of *glp-1* [*glp-1(gf)*] develop germ cell tumors when grown at 25°C (Pepper et al. 2003). Tumor development in *glp-1(ar202)* worms is known to be suppressed by the depletion of depletion of APX-1, a ligand for GLP-1 produced by the sheath cells (McGovern et al. 2009). Consistently, *apx-1(RNAi)* reduced tumor formation in *glp-1(ar202)* [*glp-1(gf)*] worms by about 30 %. By contrast, *apx-1(RNAi)* does not affect the tumor development in worms missing both GLD-1 and PUF-8. Results shown are average of triplicates; error bars represent standard deviation; and the P value was calculated using the Student's t-test.

Table S1 ***C. elegans*** **strains used in this study**

Strain	Genotype	Reference
IT60	*puf-8(zh17) unc-4(e120) / mnC1* II	(Ariz et al. 2009)
JH1500	*puf-8(ok302) unc-4(e120) / mnC1* II	(Subramaniam and Seydoux 2003)
JK3231	*puf-8(q725)* II	(Bachorik and Kimble 2005)
IT969	*puf-8(zh17) unc-4(e120) / mnC1* II; *dpy-5(e61)*	This study
IT85	*dpy-5(e61) gld-1(q485) / hT1* I; *puf-8(zh17) unc-4(e120) / mnC1* II	(Ariz 2010)
IT970	*dpy-5(e61) gld-1(q485) / hT1* I; *unc-4(e120)* II	This study
JH190	*fem-3(q20)* IV	(Barton et al. 1987)
BA606	*spe-6(hc49) unc-25(e156)* III*; eDp6(*III*;f)*	(Varkey et al. 1993)
IT995	*puf-8(zh17) unc-4(e120)/mnC1* II*; fem-3(q20)/fem-3(q20)* IV	This study
IT996	*dpy-5(e61) gld-1(q485)/hT1* I; *fem-3(q20)/fem-3(q20)* IV	This study
IT997	*dpy-5(e61) gld-1(q485)/hT1* I; *puf-8(zh17) unc-4(e120)/mnC1* II; *fem-3(q20)* IV	This study
RB798	*rrf-1(ok589)* I	International *C. elegans* Gene Knockout Consortium
IT179	*rrf-1(ok589)* I; *puf-8(zh17)* II	(Ariz 2010)
IT253	*rrf-1(ok589)* I; *puf-8(ok302)* II	(Ariz 2010)
IT958	*dpy-5(e61) gld-1(q485) / hT2* I; *puf-8(zh17) unc-4(e120) / mnC1* II; *spe-6(hc49) unc-25(e156) / hT2* III	This study
IT971	*dpy-5(e61) gld-1(q485) / hT2* I; *spe-6(hc49) unc-25(e156) / hT2* III	This study
EJ238	*mek-2(q425) unc-11(e47) I; sDp2 (I; f)*	(Church et al. 1995)
GC833	*glp-1(ar202)* III	(Pepper et al. 2003)

Supplementary references

Ariz, M., Identifying partners of PUF-8, a *C. elegans* member of the PUF family of RNA-binding proteins. Ph.D. thesis. Indian Institute of Technology, Kanpur, 2010.

Ariz, M., R. Mainpal and K. Subramaniam 2009 C. elegans RNA-binding proteins PUF-8 and MEX-3 function redundantly to promote germline stem cell mitosis. Dev. Biol. 326**:** 295-304.

Bachorik, J. L. and J. Kimble 2005 Redundant control of the Caenorhabditis elegans sperm/oocyte switch by PUF-8 and FBF-1, two distinct PUF RNA-binding proteins. Proc. Natl. Acad. Sci. U. S. A. 102**:** 10893-7.

Barton, M. K., T. B. Schedl and J. Kimble 1987 Gain-of-function mutations of fem-3, a sex-determination gene in Caenorhabditis elegans. Genetics 115**:** 107-19.

Church, D. L., K. L. Guan and E. J. Lambie 1995 Three genes of the MAP kinase cascade, mek-2, mpk-1/sur-1 and let-60 ras, are required for meiotic cell cycle progression in Caenorhabditis elegans. Development 121**:** 2525-35.

McGovern, M., R. Voutev, J. Maciejowski, A. K. Corsi and E. J. Hubbard 2009 A "latent niche" mechanism for tumor initiation. Proc. Natl. Acad. Sci. U. S. A. 106**:** 11617-22.

Pepper, A. S., D. J. Killian and E. J. Hubbard 2003 Genetic analysis of Caenorhabditis elegans glp-1 mutants suggests receptor interaction or competition. Genetics 163**:** 115-32.

Subramaniam, K. and G. Seydoux 2003 Dedifferentiation of primary spermatocytes into germ cell tumors in C. elegans lacking the pumilio-like protein PUF-8. Curr. Biol. 13**:** 134-9.

Varkey, J. P., P. L. Jansma, A. N. Minniti and S. Ward 1993 The Caenorhabditis elegans spe-6 gene is required for major sperm protein assembly and shows second site non-complementation with an unlinked deficiency. Genetics 133**:** 79-86.
