# Supplementary material for: PUF-8 Functions Redundantly with GLD-1 to Promote the Meiotic Progression of Spermatocytes in Caenorhabditis elegans
Source: G3 (Bethesda). 2015 Jun 10;5(8):1675–84. doi: 10.1534/g3.115.019521 (PMC4528324; doi:10.1534/g3.115.019521)
Supplement: Supporting Information [file supp_g3.115.019521_FileS1.pdf]

File S1

Supplementary methods

Generation of males of different genotypes

gld-1(q485), puf-8(zh17), puf-8(ok302), puf-8(q725)* and *spe-6(hc49) males were generated by mating N2 males with hermaphrodites heterozygous for the respective mutant allele and appropriate balancer (Table S1). Wild-type (heterozygous) F1 progeny males were then back-crossed with the heterozygous hermaphrodites several times until 25% of the male population were homozygous for the desired mutant allele. These males were maintained by crossing the heterozygous mutant males with the mutant hermaphrodites.

dpy-5(e61) gld-1(q485); puf-8(zh17) unc-4(e120) males were generated by mating *puf-8(zh17) unc-4(e120) / mnC1* males with IT85 [*dpy-5(e61) gld-1(q485) / hT1 I; puf-8(zh17) unc-4(e120) / mnC1* II] hermaphrodites. Wild-type F1 progeny males were then back-crossed with the mutant hermaphrodites and this process was repeated until 25% of the male population in the plate were homozygous for the desired mutant allele. Genotypes of homozygous mutant males were confirmed based on the phenotypes of the marker mutations linked to *gld-1(q485)* and *puf-8(zh17)* alleles initially and then by the tumorous germline phenotype of *gld-1(q485); puf-8(zh17)*. These males were maintained by crossing the heterozygous males with the IT85 hermaphrodites.

rrf-1(ok589); puf-8(zh17)* and *rrf-1(ok589); puf-8(ok302) males were generated by mating *puf-8(zh17) unc-4(e120) / mnC1* males and *puf-8(ok302) unc-4(e120) / mnC1* males with IT179 [*rrf-1(ok589); puf-8(zh17)*] and IT253 [*rrf-1(ok589); puf-8(ok302)*] hermaphrodites, respectively. Wild-type F1 progeny males were then back-crossed with the respective mutant hermaphrodites and this process was repeated several times to obtain homozygous mutant males. *rrf-1(ok589)* mutation was detected by PCR and the *puf-8* allele by the phenotype of the marker mutation and also by checking for the *puf-8* phenotype at 25ºC.

gld-1(q485); spe-6(hc49) males were generated by mating *dpy-5(e61) gld-1(q485) / hT1* males with IT971 [*dpy-5(e61) gld-1(q485) / hT2* I*; spe-6(hc49) unc-25(e156) / hT2* III] hermaphrodites. Wild-type F1 progeny males were then back-crossed with the respective mutant hermaphrodites and this process was repeated several times to obtain homozygous mutant males. The genotypes of homozygous mutant males were confirmed by the phenotypes of marker mutations linked to *gld-1(q485)* and *spe-6(hc49),* and also by checking for the phenotype of *gld-1(q485); puf-8(zh17)* hermaphrodites.

gld-1(q485); puf-8(zh17); spe-6(hc49) males were generated by mating *puf-8(zh17) / mC6g* males with IT958 [*dpy-5(e61) gld-1(q485) / hT2* I*; puf-8(zh17) unc-4(e120) / mnC1* II; *spe-6(hc49) unc-25(e156) / hT2* III] hermaphrodites. GFP-positive F1 progeny males were selected using a fluorescence stereomicroscope and mated with IT958 hermaphrodites and this process was repeated several times. Non-GFP males that displayed the dumpy and uncoordinated phenotypes were selected to check for the phenotype of *gld-1(q485); puf-8(zh17); spe-6(hc49)*.
