# Supplementary material for: PUF-8 Functions Redundantly with GLD-1 to Promote the Meiotic Progression of Spermatocytes in Caenorhabditis elegans
Source: G3 (Bethesda). 2015 Jun 10;5(8):1675–84. doi: 10.1534/g3.115.019521 (PMC4528324; doi:10.1534/g3.115.019521)
Supplement: Supporting Information [file supp_g3.115.019521_FigureS1.pdf]

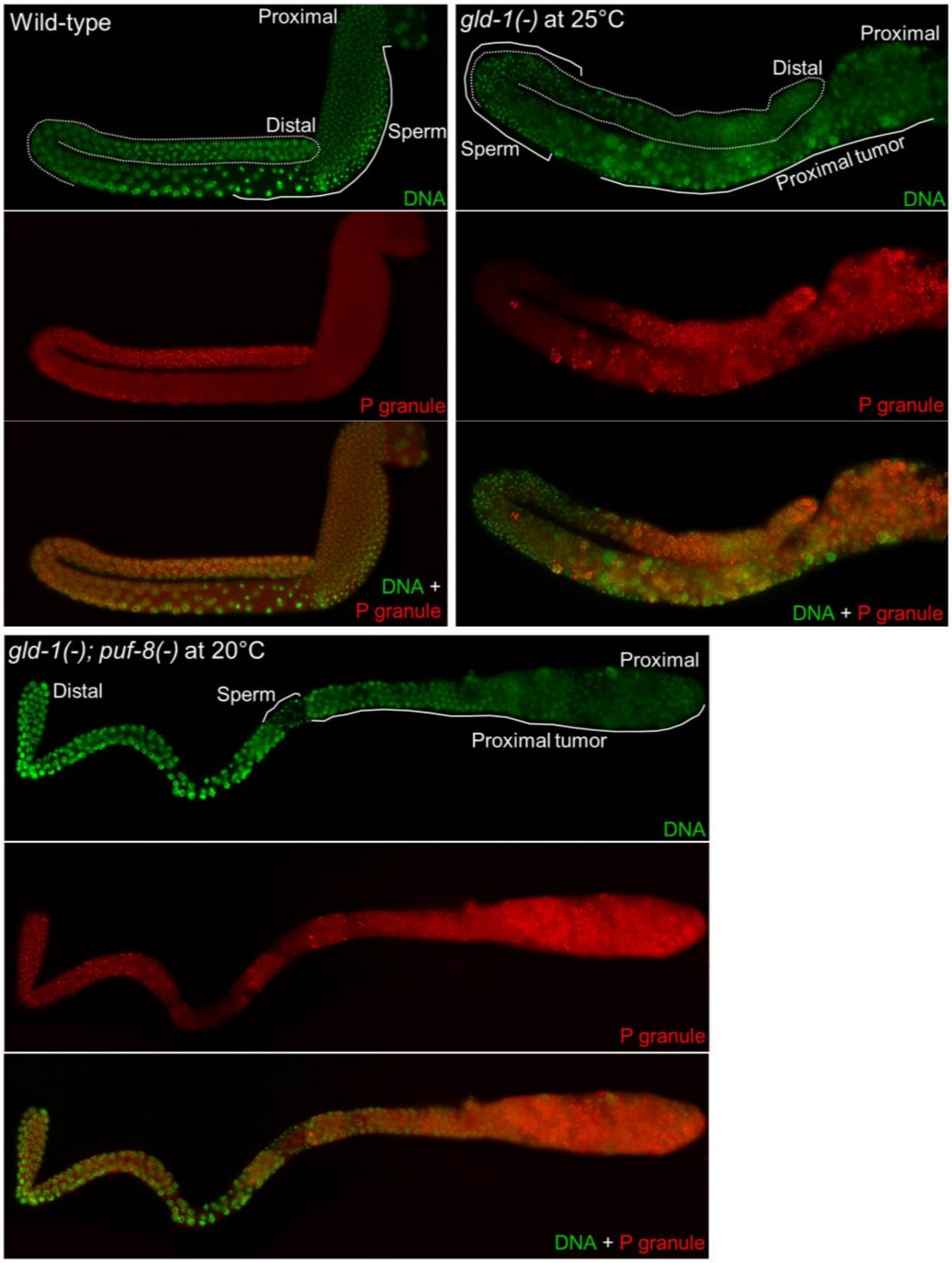

Figure S1 The germ cell-specific P granules are present in the tumor cells observed in the germlines of *gld-1(-)* and *gld-1(-); puf-8(-)* males. Dissected gonads of the indicated genotypes stained with anti-P granule antibodies and DAPI. While the P granules are not seen in the sperm present in the proximal part of wild-type gonads, they are readily visible in the proliferating cells present in the same region of the *gld-1(-)* and *gld-1(-); puf-8(-)* germlines.
