# Supplementary material for: PUF-8 Functions Redundantly with GLD-1 to Promote the Meiotic Progression of Spermatocytes in Caenorhabditis elegans
Source: G3 (Bethesda). 2015 Jun 10;5(8):1675–84. doi: 10.1534/g3.115.019521 (PMC4528324; doi:10.1534/g3.115.019521)
Supplement: Supporting Information [file supp_g3.115.019521_FigureS2.pdf]

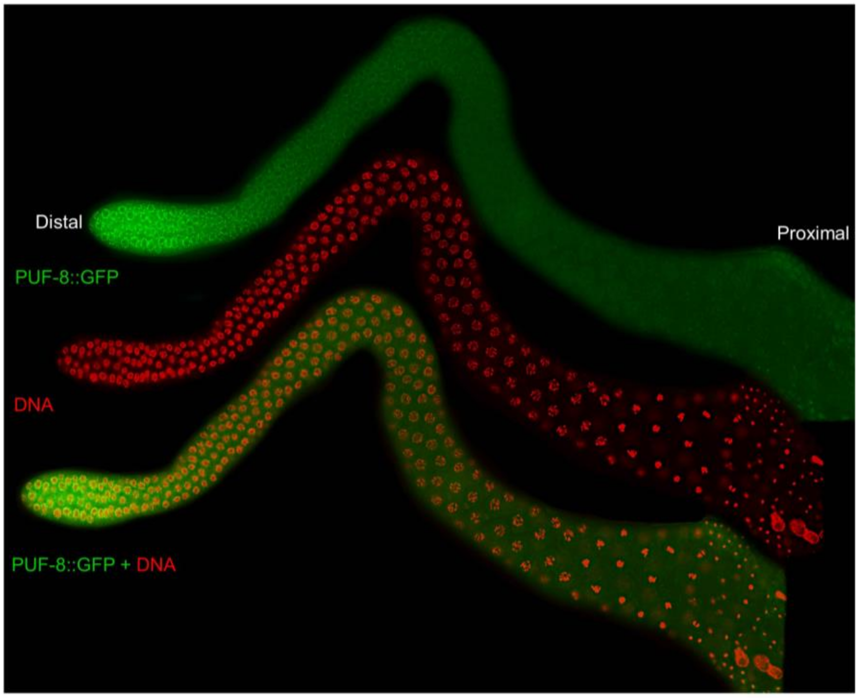

Figure S2 Expression pattern of PUF-8::GFP in male germlines. Dissected germline of a male carrying the kpIs[pMP15] transgene. This transgene expresses PUF-8::GFP fusion under the control of *puf-8* promoter and *puf-8* 3' UTR (Ariz et al. 2009). Strong expression of PUF-8::GFP (green) is seen in the distal germline, where PUF-8::GFP localization on perinuclear P granules is noticeable. DNA has been visualized by staining with Hoechst stain (red).
