# Supplementary material for: PUF-8 Functions Redundantly with GLD-1 to Promote the Meiotic Progression of Spermatocytes in Caenorhabditis elegans
Source: G3 (Bethesda). 2015 Jun 10;5(8):1675–84. doi: 10.1534/g3.115.019521 (PMC4528324; doi:10.1534/g3.115.019521)
Supplement: Supporting Information [file supp_g3.115.019521_FigureS3.pdf]

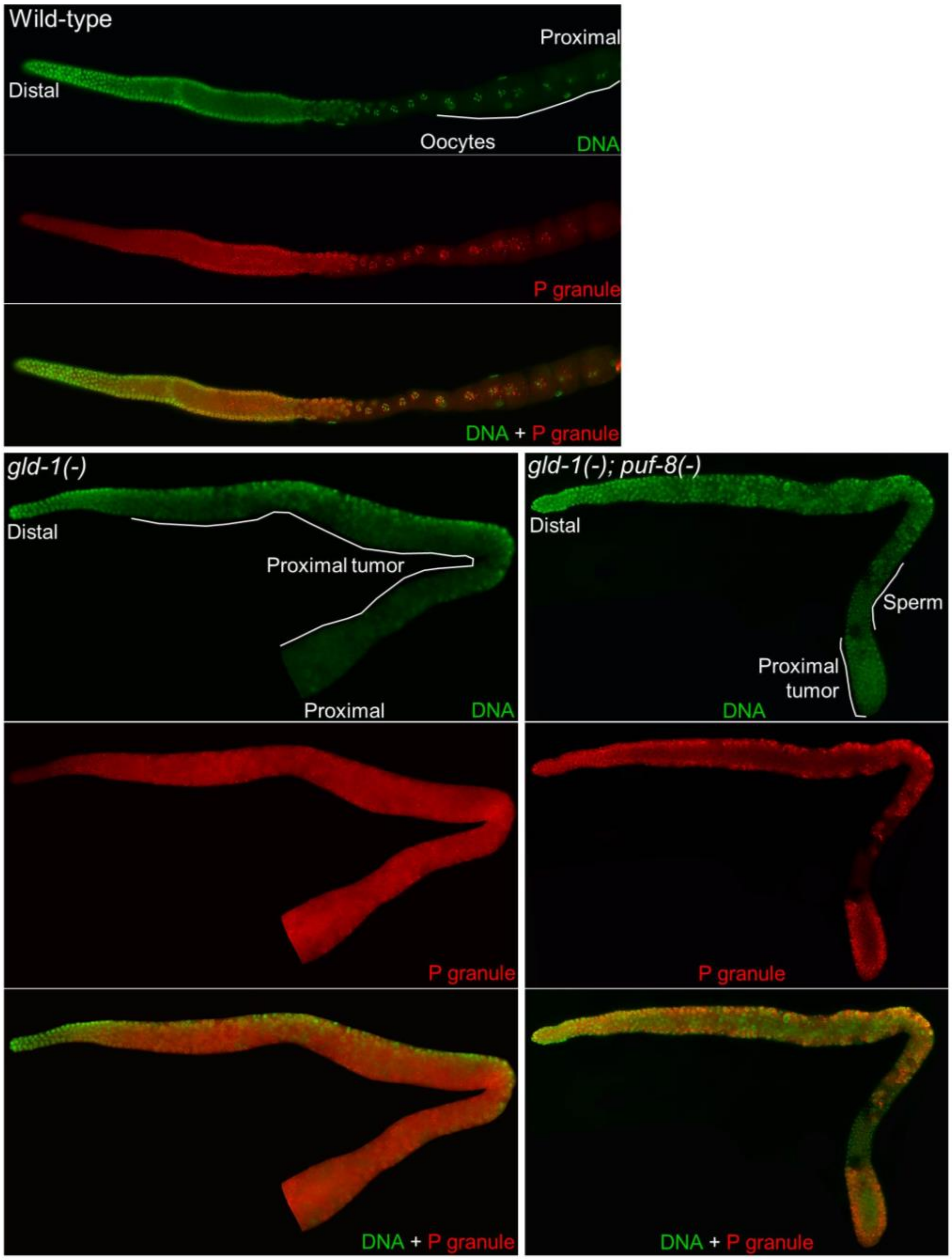

Figure S3 Tumor cells of *gld-1(-)* and *gld-1(-); puf-8(-)* hermaphrodites contain P granules. Dissected gonads of the indicated genotypes stained with anti-P granule antibodies and DAPI. In the wild-type germline, the P granules are seen in all cells including the developing oocytes. Similarly, the proximal tumor cells of *gld-1(-)* and *gld-1(-)*; *puf-8(-)* germlines as well stain positively for the germ cell-specific P granules. However, P granules are not present in the sperm seen in the *gld-1(-); puf-8(-)* germline; absence of P granules in sperm has been observed in the wild-type as well (Subramaniam and Seydoux 2003).
