# Supplementary material for: PUF-8 Functions Redundantly with GLD-1 to Promote the Meiotic Progression of Spermatocytes in Caenorhabditis elegans
Source: G3 (Bethesda). 2015 Jun 10;5(8):1675–84. doi: 10.1534/g3.115.019521 (PMC4528324; doi:10.1534/g3.115.019521)
Supplement: Supporting Information [file supp_g3.115.019521_FigureS4.pdf]

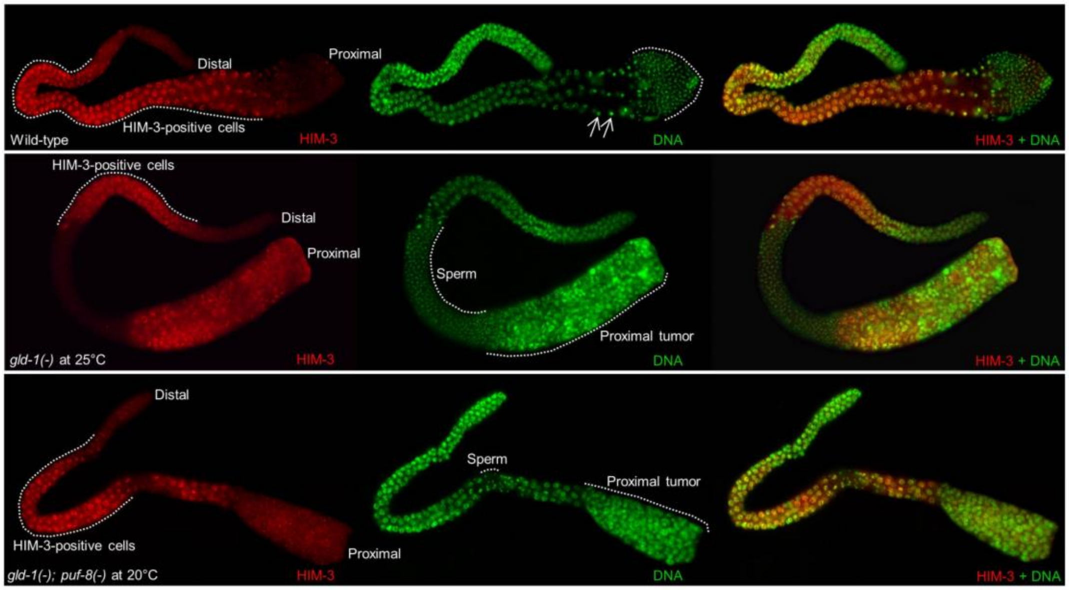

Figure S4 Meiotic entry is unaffected in *gld-1(-)* and *gld-1(-); puf-8(-)* males grown at 25°C. Gonads have been extruded out of males raised at 25°C and stained for the HIM-3 meiotic marker (red) and DAPI (green). Region of the germline containing HIM-3-positive cells have been indicated by a dashed white line in images shown on the left panel. Proximal tumors are outlined in images shown on the middle panel.
