# Supplementary material for: PUF-8 Functions Redundantly with GLD-1 to Promote the Meiotic Progression of Spermatocytes in Caenorhabditis elegans
Source: G3 (Bethesda). 2015 Jun 10;5(8):1675–84. doi: 10.1534/g3.115.019521 (PMC4528324; doi:10.1534/g3.115.019521)
Supplement: Supporting Information [file supp_g3.115.019521_FigureS5.pdf]

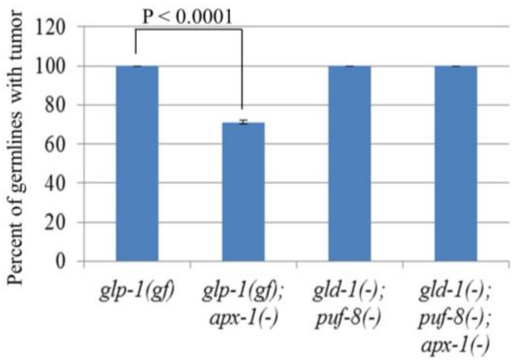


**Figure S5** Proximal proliferation in the *gld-1(-); puf-8(-)* mutants is not dependent on latent niche signaling. Bar graph showing the effect of APX-1 depletion on tumor development. Worms homozygous for the *ar202* allele of *glp-1* [*glp-1(gf)*] develop germ cell tumors when grown at 25°C (Pepper et al. 2003). Tumor development in *glp-1(ar202)* worms is known to be suppressed by the depletion of depletion of APX-1, a ligand for GLP-1 produced by the sheath cells (McGovern et al. 2009). Consistently, *apx-1(RNAi)* reduced tumor formation in *glp-1(ar202)* [*glp-1(gf)*] worms by about 30 %. By contrast, *apx-1(RNAi)* does not affect the tumor development in worms missing both GLD-1 and PUF-8. Results shown are average of triplicates; error bars represent standard deviation; and the P value was calculated using the Student's t-test.
