# Supplementary material for: PUF-8 Functions Redundantly with GLD-1 to Promote the Meiotic Progression of Spermatocytes in Caenorhabditis elegans
Source: G3 (Bethesda). 2015 Jun 10;5(8):1675–84. doi: 10.1534/g3.115.019521 (PMC4528324; doi:10.1534/g3.115.019521)
Supplement: Supporting Information [file supp_g3.115.019521_TableS1.pdf]

Table S1 ***C. elegans*** **strains used in this study**

Strain	Genotype	Reference
IT60	*puf-8(zh17) unc-4(e120) / mnC1* II	(Ariz et al. 2009)
JH1500	*puf-8(ok302) unc-4(e120) / mnC1* II	(Subramaniam and Seydoux 2003)
JK3231	*puf-8(q725)* II	(Bachorik and Kimble 2005)
IT969	*puf-8(zh17) unc-4(e120) / mnC1* II; *dpy-5(e61)*	This study
IT85	*dpy-5(e61) gld-1(q485) / hT1* I; *puf-8(zh17) unc-4(e120) / mnC1* II	(Ariz 2010)
IT970	*dpy-5(e61) gld-1(q485) / hT1* I; *unc-4(e120)* II	This study
JH190	*fem-3(q20)* IV	(Barton et al. 1987)
BA606	*spe-6(hc49) unc-25(e156)* III*; eDp6(*III*;f)*	(Varkey et al. 1993)
IT995	*puf-8(zh17) unc-4(e120)/mnC1* II*; fem-3(q20)/fem-3(q20)* IV	This study
IT996	*dpy-5(e61) gld-1(q485)/hT1* I*; fem-3(q20)/fem-3(q20)* IV	This study
IT997	*dpy-5(e61) gld-1(q485)/hT1* I; *puf-8(zh17) unc-4(e120)/mnC1* II; *fem-3(q20)* IV	This study
RB798	*rrf-1(ok589)* I	International *C. elegans* Gene Knockout Consortium
IT179	*rrf-1(ok589)* I; *puf-8(zh17)* II	(Ariz 2010)
IT253	*rrf-1(ok589)* I; *puf-8(ok302)* II	(Ariz 2010)
IT958	*dpy-5(e61) gld-1(q485) / hT2* I; *puf-8(zh17) unc-4(e120) / mnC1* II; *spe-6(hc49) unc-25(e156) / hT2* III	This study
IT971	*dpy-5(e61) gld-1(q485) / hT2* I; *spe-6(hc49) unc-25(e156) / hT2* III	This study
EJ238	*mek-2(q425) unc-11(e47) I; sDp2 (I; f)*	(Church et al. 1995)
GC833	*glp-1(ar202)* III	(Pepper et al. 2003)
